# [Corrigendum] Combination of the FGFR4 inhibitor PD173074 and 5-fluorouracil reduces proliferation and promotes apoptosis in gastric cancer

**DOI:** 10.3892/or.2026.9215

**Published:** 2026-10-26

**Authors:** Yan-Wei Ye, Shuang Hu, Ying-Qiang Shi, Xie-Fu Zhang, Ye Zhou, Chun-Lin Zhao, Guo-Jun Wang, Jian-Guo Wen, Hong Zong

Oncol Rep 30: 2777-2784, 2013; DOI: 10.3892/or.2013.2796

Following the publication of the above article, and an Expression of concern statement (doi: 10.3892/or.2025.9004) that was published after a concerned reader drew the Editor's attention to the fact that, for the western blot data shown in Figs. 1E and 4, the FGFR blots and the control GAPDH blots appeared to be strikingly similar, suggesting that the same data had been included in these figures even though the results from differently performed experiments were intended to have been portrayed, the authors have now responded to the Editorial Office to address this issue. Owing to the similarities in these data, the authors have repeated the experiments shown in Fig. 1E, and the revised version of Fig. 1 including this figure part is shown below. Note that the revisions made to this figure do not affect the overall conclusions reported in the paper. The authors are grateful to the Editor of *Oncology Reports* for allowing them the opportunity to publish this Corrigendum, and all the authors agree with its publication; furthermore, they apologize to the readership for any inconvenience caused.

## Figures and Tables

**Figure 1.**
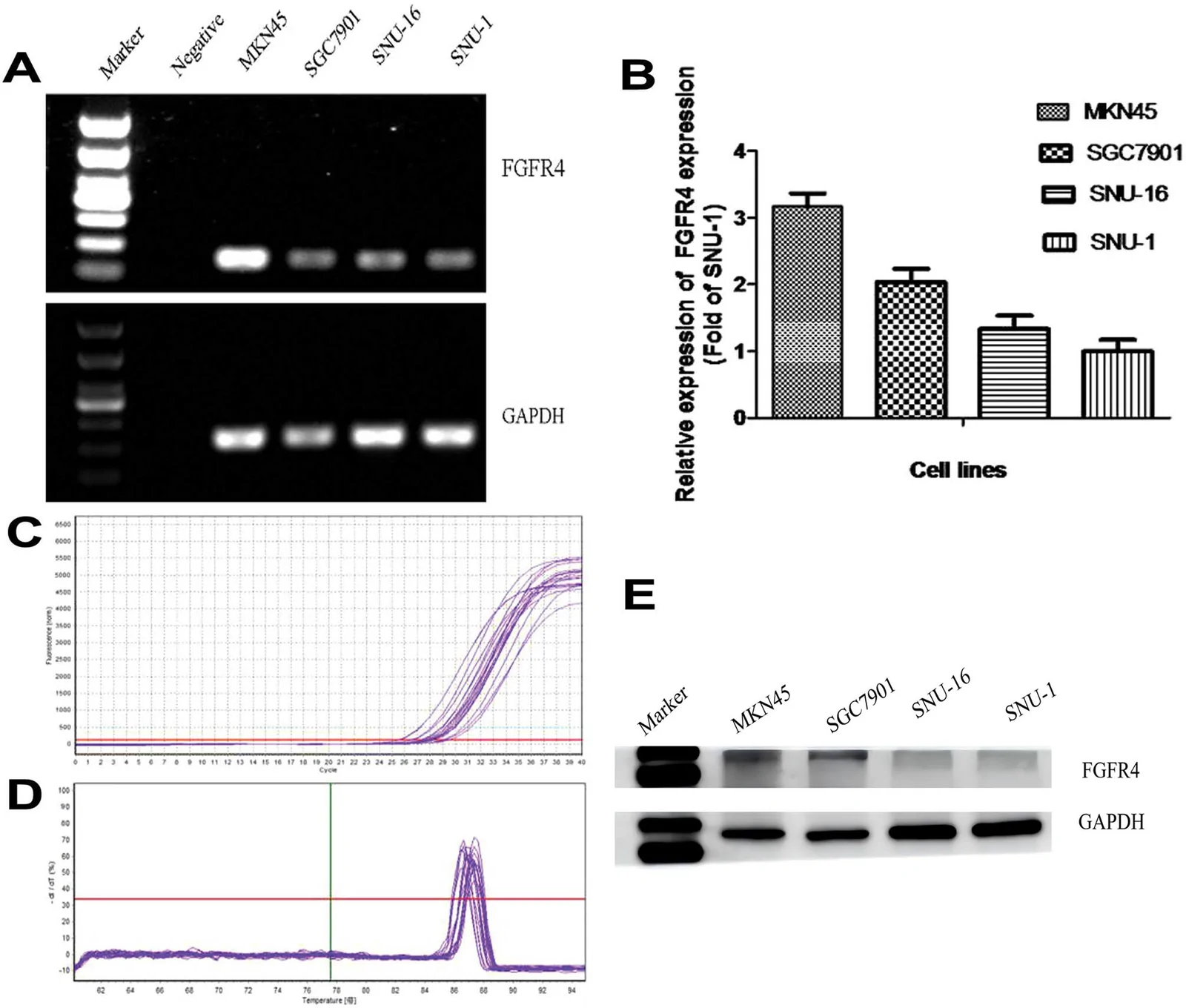
Expression of FGFR4 mRNA and protein in gastric cancer cell lines, MKN45, SGC7901, SNU-1 and SNU-16. Expression of FGFR4 mRNA in the different GC cell lines was revealed by (A) RT-PCR and (B) quantitative real-time PCR. (C and D) Illustrated melting curve and quantification curve of FGFR4, respectively. (E) Expression of FGFR4 protein in the different GC cell lines was assessed by western blot analysis; GAPDH served as the loading control. Expression of FGFR4 mRNA and protein in MKN45 cells was the highest among the 4 GC cell lines.

